# Determination of the Mutant Selection Window and Evaluation of the Killing of *Mycoplasma gallisepticum* by Danofloxacin, Doxycycline, Tilmicosin, Tylvalosin and Valnemulin

**DOI:** 10.1371/journal.pone.0169134

**Published:** 2017-01-04

**Authors:** Nan Zhang, Xiaomei Ye, Yuzhi Wu, Zilong Huang, Xiaoyan Gu, Qinren Cai, Xiangguang Shen, Hongxia Jiang, Huanzhong Ding

**Affiliations:** 1 Guangdong Key Laboratory for Veterinary Drug Development and Safety evaluation, South China Agricultural University, Guangzhou, China; 2 Technical Center of Zhuhai Entry-Exit Inspection and Quarantine Bureau, Zhuhai, China; Miami University, UNITED STATES

## Abstract

*Mycoplasma gallisepticum* is a common etiological cause of a chronic respiratory disease in chickens; its increasing antimicrobial resistance compromises the use of tetracyclines, macrolides and quinolones in the farm environment. Mutant selection window (MSW) determination was used to investigate the propensity for future resistance induction by danofloxacin, doxycycline, tilmicosin, tylvalosin and valnemulin. Killing of *M*. *gallisepticum* strain S6 by these antimicrobials was also studied by incubating *M*. *gallisepticum* into medium containing the compounds at the minimal concentration that inhibits colony formation by 99% (MIC_99_) and the mutant prevention concentration (MPC). Based on the morphology and colony numbers of *M*. *gallisepticum* on agar plates, the four kinds of sera in the order of the applicability for culturing *M*. *gallisepticum* were swine serum > horse serum > bovine serum > mixed serum. The MPC/MIC_99_ values for each agent were as follows: danofloxacin > tilmicosin > tylvalosin > doxycycline > valnemulin. MPC generated more rapid and greater magnitude killing than MIC_99_ against *M*. *gallisepticum*. Under exposure of 10^5^–10^9^ CFU/mL at MPC drug levels, valnemulin had the slowest rate of reduction in viable organisms and danofloxacin had the highest rate of reduction.

## Introduction

The ‘mutant selection window’ (MSW) hypothesis is a novel concept described by Zhao and Drlica[1], which postulates that a drug concentration zone exists in which resistant mutants are selectively amplified, generating reduced drug susceptibility. The lower boundary of the MSW is the lowest concentration that blocks the growth of the majority of susceptible cells. This is often approximated by the minimum inhibitory concentration (MIC) or the minimal concentration that inhibits colony formation by 99% (MIC_99_). The upper boundary is the minimum concentration that inhibits growth of the least-susceptible single-step mutant subpopulation, the mutant prevention concentration (MPC). Keeping drug concentrations above MPC is expected to restrict the emergence of resistance[2]. This hypothesis has been validated by *in vitro* and *in vivo* experiments[3–6]. To date, MPC studies using fluoroquinolones, tetracyclines, macrolides and β- lactams have been applied in a variety of microorganisms including *Staphylococcus aureus*, *Escherichia coli*, *Mycobacterium tuberculosis*, *Mannheimia haemolytica* and *Streptococcus pneumoniae* [7–11].

*Mycoplasma gallisepticum* is an important pathogen in poultry that causes chronic respiratory disease in chickens and sinusitis in turkeys. The primary clinical signs of *M*. *gallisepticum* infection include ocular and nasal discharge, coughing, sneezing, tracheal rales and mild conjunctivitis [12]. These infections can lead to considerable economic losses in the poultry industry due to increased embryo mortality, as well as reduced weight gain and egg production in commercial birds. Many antimicrobial agents, such as fluoroquinolones, tetracyclines, macrolides, and pleuromutilins have *in vitro* activity against mycoplasmas[13, 14]. However, long-term and sometimes unreasonable use such as inadequate and indiscriminate application of antimicrobial agents has resulted in the emergence and spreading of *M*. *gallisepticum* resistance to the drugs. While *M*. *gallisepticum* is minute in size and completely lacks a bacterial cell wall, it should be cultivated on specially formulated media. Quantitative cultures by viable count estimation (CFU determination) are complicated by these strict nutritional conditions. As such, there have been few reported studies about *M*. *gallisepticum*; the current investigation represents the first attempt to determine MPC of antimicrobial agents against *M*. *gallisepticum*.

The purpose of this study was to determine the MSW of danofloxacin, doxycycline, tilmicosin, tylvalosin and valnemulin against *M*. *gallisepticum* strain S6 *in vitro*. Firstly, we compared two methods for MIC determination using three inoculum sizes. The MIC_99_ and MPC of the antimicrobials were determined using the agar dilution method. *In vitro* killing for the five antimicrobial agents against *M*. *gallisepticum* strain S6 ranging from 10^5^ CFU/mL to 10^9^ CFU/mL was also determined using the measured MIC_99_ and MPC concentrations for each antibiotic. This study suggested that the MSW existed in danofloxacin, doxycycline, tilmicosin, tylvalosin and valnemulin against *M*. *gallisepticum in vitro*.

## Materials and Methods

*M*. *gallisepticum* standard strain S6 was obtained as a freeze-dried powder from the China Institute of Veterinary Drug Control (Beijing) and stored at -80°C. Broth medium base, NADH and cysteine were obtained from Qingdao Hope Biological Technology. Sterile swine serum, bovine serum and horse serum were purchased from Guangzhou Ruite Biological Technology. The initial pH of the medium was adjusted to pH 7.8±0.1 with 1 M NaOH, and solid media was supplemented with 1% agar.

Danofloxacin, doxycycline, tilmicosin, tylvalosin and valnemulin were kindly supplied by Guangdong Dahuanong Animal Health Products. Immediately prior to each experiment, the antibacterial agents were dissolved in Milli-Q water and sterilized by filtration. Fresh stock solutions of the antibacterial agents were prepared at 2,560 mg/L.

### The ability of different types of animal serum to support *M. gallisepticum* growth on agar plates

In order to optimize the suitable growth condition of *M*. *gallisepticum* on solid agar plates, different mixtures of inactivated 10% animal sera were added to solid media. These were 10% swine serum, 10% bovine serum, 10% horse serum and 10% mixed serum (3.33% swine, 3.33% bovine and 3.33% horse serum). The prepared plates were uniformly inoculated with 10 μL-drops of appropriately diluted broth culture of *M*. *gallisepticum*. After the inoculum fluid was absorbed by the agar, the plates were incubated at least for 7 days in a 37°C, 5% CO_2_ humidified incubator. An inverted microscope was used for colony counting. The number of CFU per 10 μL of inoculum was the mean count from three droplets. All experiments were performed in triplicate and conducted on different days to ensure data reproducibility.

### Determination of minimum inhibitory concentrations

A modified MIC assay method was used to determine MIC as described by Tanner and Wu [15]. *M*. *gallisepticum* exponential-phase cultures were diluted with growth medium to 10^5^, 10^6^ and 10^7^ CFU/mL. Overlapping sets of twofold serial dilutions were used to improve accuracy. For example, danofloxacin, doxycycline, tilmicosin and tylvalosin concentration ranges were 0.0063–0.40 mg/L and 0.0047–0.30 mg/L. For valnemulin, 0.00063–0.040 mg/L and 0.00047–0.030 mg/L were used. Aliquots of 0.10 mL antibiotic solution were added to an equal volume of an exponential-phase culture together in a 96-well plate. Sterility control (sterile broth at pH 7.8), growth control (inoculum in absence of antimicrobials) and end-point control (blank medium at pH 6.8) were included. Plates were cultured in a 37°C, 5% CO_2_ humidified incubator after being sealed with parafilm. Color changes were monitored until the color of the growth control was the same as that of the end-point control. The MIC was determined as the minimal concentration of antibacterial agent that resulted in no color change.

The agar dilution technique for MIC determination followed that described by Hannan[16]. Solutions containing twenty times (20 ×) the MIC concentrations were used for making agar plates of two-fold overlapping dilutions of: 0.50–32.00 mg/L and 0.38–24.00 mg/L for danofloxacin, doxycycline, tilmicosin, and tylvalosin; 0.050–3.20 mg/L and 0.038–2.40 mg/L for valnemulin. Ten μL of a *M*. *gallisepticum* culture (10^5^–10^7^ CFU) was applied to the plates followed by incubation for at least 7 days as described above. Growth control plates lacking antimicrobials were included in each test. The MIC was determined as the minimal concentration of antibacterial agent that resulted in no growth on the agar plate.

For MIC, MIC_99_ and MPC tests on solid medium, the drug was incorporated into the agar plates at the final required concentrations, and the final volume of medium per plate was 20 mL. The 7 cm diameter circle of an agar plate showed a great advantage over other plates because the agar thickness provided sufficient nutrition to allow the slow growing mycoplasma to form clearly visible colonies.

### Determination of minimum inhibitory concentration that inhibited growth of 99% of the inoculated cells

Measurements of MIC_99_ were based on the method reported by Lu et al[17] with modifications. The drug dosages were prepared by linear decreasing dilutions based on the MIC value. About 10% per sequential dilution decrease ranged from 1 × MIC to 0.5 × MIC. A 10^7^ CFU/mL cell suspension from logarithmic growth phase cells was diluted in a 10-fold series and 10 μL of each diluted suspension was inoculated onto the plates. Plates were cultured in a 37°C, 5% CO_2_ humidified incubator for at least 7 days. Colony numbers between 30 and 300 were counted. The fraction of colonies recovered was plotted against drug concentration to determine the MIC_99_ by interpolation.

### Measurement of the mutant prevention concentration

MPC was defined as the lowest drug concentration that prevented bacterial colony formation from a culture containing 10^10^ CFU bacteria. Initially we tested three different pretreatment methods for enriching *M*. *gallisepticum*. Cultures were first grown to a final concentration of ∼3 × 10^10^ CFU/mL.

Different methods were used in this study. With ‘Method 1’, 5 mL of logarithmic phase culture was added into 50 mL broth and incubated for 36 h. The suspension was concentrated by centrifugation (4,000 × g for 20 min) and the cell pellet was suspended in 1 ml media for cell counting. In ‘Method 2’, the incubation steps were the same as ‘Method 1’, but 1 ml of suspended culture was supplemented with 100 ml of broth and incubated for 72 h. The cells were concentrated by centrifugation and suspended in 0.5 ml media for counting. For ‘Method 3’, 20 mL of a stationary growth phase culture was added to 180 mL broth and incubated for 36 h. The culture was packaged into 50-ml tube and concentrated by centrifugation (4,000 × g for 20 min), then the cells were suspended in 1 mL media. Four of these 1 mL aliquots were combined and concentrated by centrifugation (6,000 × g for 20 min). The cells were suspended in 0.5 ml of culture medium for counting.

In preliminary experiments, different centrifugation protocols using varying speeds (2000, 3000, 4000, 5000, 6,000 g) and centrifugation times (5, 10, 15, 20, 25, 30 min) for enriching *M*. *gallisepticum* were compared (data not shown). A higher centrifugal efficiency was obtained using 4,000 g for 20 min followed by a second centrifugation at 6,000 g for 20 min. With higher speeds we found that cell membrane rupture occurred, while at very low speeds the cells did not settle completely.

After the pretreatment for cell enrichment, 100 μL of the suspensions were plated on agar plates containing various antibiotic concentrations. The plates were incubated in a 37°C, 5% CO_2_ humidified incubator for 7–10 days. The preliminary MPC was recorded as the lowest antibiotic concentration that allowed no colony formation. This determination of MPC was followed by a second measurement that utilized linear drug concentration decrements (about 20% per sequential decrease). The MPC was recorded as the lowest drug concentration preventing growth.

### Time–killing studies

The *in vitro* time–killing studies were described elsewhere[18]. Briefly, *M*. *gallisepticum* was inoculated into 10 mL medium containing antibiotic concentrations based on the measured MIC_99_ or MPC drug concentrations. The inocula were adjusted from 10^5^ CFU/mL to 10^9^ CFU/mL. Cultures were incubated at 37°C with 5% CO_2_ for 48 h. Aliquots of 100 μL from each culture were collected at 0, 3, 6, 9, 12, 24, 36 and 48 h. Viable cell numbers were determined *via* 10-fold serial dilutions and plating 10 μL of each diluted culture on drug-free agar plates. Growth control (*M*. *gallisepticum* culture in the absence of drugs) and sterility control (10 ml of medium at pH 7.8) were also included. Plates were incubated at least 7 days in a 37°C, 5% CO_2_ humidified incubator. As previously reported, a reduction of ≥ 3 log10 is associated with mycoplasmacidal activity and reductions ≤ 2 log10 are defined as mycoplasmastatic activity[19].

## Results

### The ability of different types of animal sera to support *M. gallisepticum* growth on agar plates

The growth condition of *M*. *gallisepticum* on the agar plates supplemented with different kinds of inactivated animal sera is summarized in Table 1. The morphology of *M*. *gallisepticum* on agar plates was also observed using an inverted microscope. Even though there was little difference in colony numbers between the four kinds of the serum, the largest colonies (solid media containing swine serum) were approximately twice the size of the smallest (solid media containing mixed serum). The colony sizes also varied and contained different shapes and sizes with mixed serum. However, it was difficult to accurately count the colonies with small size.

**Table 1:** Effects of different serum types on *M*. *gallisepticum* growth on agar plates.

	Mean value (log CFU/mL)	SD	CV (%)	P
Swine serum	8.75	0.041	0.47	1.00
Horse serum	8.67	0.14	1.57	0.53
Bovine swine	8.48	0.052	0.62	0.85
Mixed serum	8.64	0.13	1.52	0.17

The experiment were performed in triplicate and conducted on three days.

Mean value: The data are the average of the nine replicates.

SD: Standard deviation of the nine replicates.

CV: Coefficient of variation of the nine replicates.

P: The statistical significance of the nine replicates which were calculated using the SPASS software 19.0. P < 0.05 indicates there was a significant difference between the data.

We found that the size and number of *M*. *gallisepticum* colonies reached a maximum (8.75 log10 CFU) on solid media containing swine serum. The highest titers on agar plates with 10% inactivated swine serum also gave rise to colonies of uniform size. There were no significant difference in colony size and number between the solid media containing swine and horse serum. Nonetheless, varying sizes of the colonies were observed from the different batches of horse serum.

The standard deviation (SD = 0.041) and coefficient of variation (CV = 0.47%) were the smallest in the solid media containing swine serum. Based on the morphology and colony numbers of *M*. *gallisepticum* on agar plates, the four kinds of sera in the order of the applicability for culturing *M*. *gallisepticum* were swine serum > horse serum > bovine serum > mixed serum.

### MIC determinations

**Fig 1:**
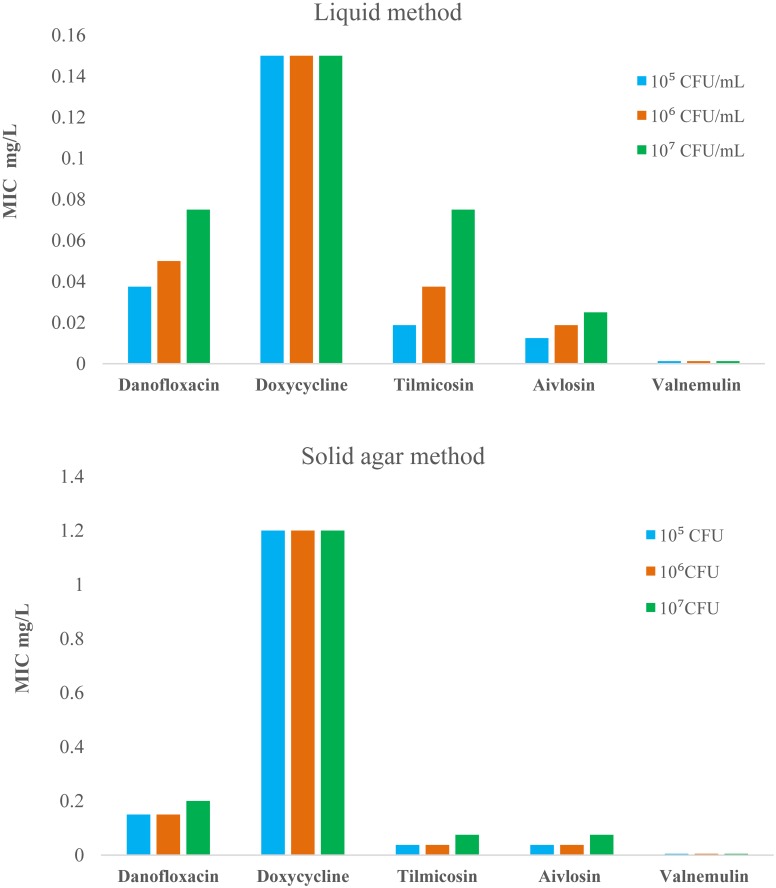
Minimum inhibitory concentrations for danofloxacin, doxycycline, tilmicosin, tylvalosin and valnemulin against *M*. *gallisepticum* strain S6 in artificial medium using the liquid and solid agar methods with inoculum sizes of 10^5^,10^6^ and 10^7^ CFU/mL.

The MIC values determined by liquid MIC method and solid agar MIC method are presented in Fig 1. These MIC values with liquid MIC method were 4-fold lower (danofloxacin), 8-fold lower (doxycycline), 2-fold lower (tilmicosin), 3-fold lower (tylvalosin), and 4-fold lower (valnemulin) than those determined by the solid agar method at an equivalent inoculum size of 10^5^ CFU/mL. A larger inoculum size (10^7^ CFU/mL) resulted in increased MICs for danofloxacin (2-fold), tilmicosin (4-fold) and tylvalosin (2-fold) than those from the lower inoculum size (10^5^ CFU/mL) using the liquid MIC method. However, for doxycycline and valnemulin, similar MIC values were obtained from the high and low inoculum sizes. Moreover, valnemulin was found to exhibit marginally superior activity against *M*. *gallisepticum* because the MIC values were minimum for the liquid and solid agar methods.

### Pretreatment methods for enriching *M. gallisepticum*

The results of three different pretreatment methods to enrich *M*. *gallisepticum* are listed in Table 2. The number of colony-forming units increased significantly and reached to maximum by the third enrichment method. The mean number of colonies (10.54 Log10 CFU) was obtained by this reliable and practical method. The smallest SD and CV (SD = 0.11, CV = 1.06%) were also observed by the third method and there was no significant difference (P = 0.073) between the data. ‘Method 3’ that took 36 h was found to be time-effective, compared to ‘the method 2’ that took 110 h.

**Table 2:** The results of three different pretreatment methods for *M*. *gallisepticum* enrichment.

Method	Mean value (log CFU/mL)	SD	CV (%)	P
1	9.43	0.19	2.04	0.49
2	9.57	0.27	2.85	0.64
3	10.54	0.11	1.06	0.073

The experiment were performed in triplicate and conducted on three days.

Mean value: The data are the average of the nine replicates.

SD: Standard deviation of the nine replicates.

CV: Coefficient of variation of the nine replicates.

P: The statistical significance of the nine replicates which were calculated using the SPASS software 19.0. P < 0.05 indicates there was a significant difference between the data.

### MIC_99_, MPC and Selection Index (SI)

The ratio of MPC to MIC_99_ (selection index, SI) reflects the ability of an antibacterial agent to select resistant mutants. In all instances in this study, the MPC values were higher than MIC values. MIC_99_ for all drugs ranged from 0.0038 to 1.10 mg/L and MPC ranged from 0.0051 to 2.46 mg/L.

The SI values for the antimicrobials are shown in Table 3. The MPC/MIC_99_ ratios arranged in order from high to low were as follows: danofloxacin (quinolones) > tilmicosin (macrolides) > tylvalosin (macrolides) > doxycycline (tetracyclines) > valnemulin (pleuromutilins). Valnemulin had the lowest selection index and its MPC was about 1.35 times higher than MIC_99_ value. On the contrary, danofloxacin had the maximum selection index and its MPC was about 24 times greater than its MIC_99_ value (Table 3).

**Table 3:** Comparison of MIC_99_, MIC, MPC and selection indices for five antimicrobial agents tested against *M*. *gallisepticum* strain S6.

	MIC_99_ (mg/L)	MIC(mg/L)	MPC(mg/L)	SI
Danofloxacin	0.10	0.15	2.40	24.00
Doxycycline	1.10	1.20	2.46	2. 24
Tilmicosin	0.027	0.038	0.15	5.56
Tylvalosin	0.030	0.038	0.15	5.12
Valnemulin	0.0038	0.0050	0.0051	1.35

MIC_99_: minimal concentrations inhibiting colony formation by 99%

MIC: minimum inhibitory concentrations

MPC: mutant prevention concentrations

SI: selection indices, the ratio of MPC to MIC_99_

### *In vitro* killing studies

Bactericidal activities of compounds were tested with three different inoculum sizes. The reduction of *M*. *gallisepticum* counts for three different inoculum sizes based on the measured MIC_99_ and MPC concentrations are listed in Tables 4 and 5.

**Table 4:** The reduction of *M*. *gallisepticum* growth for three different inoculum sizes based on the measured MIC_99_ concentrations.

Inoculum size (CFU/mL)	Time (h)	Danofloxacin (log10 CFU/mL)	Doxycycline (log10 CFU/mL)	Tilmicosin (log10 CFU/mL)	Tylvalosin (log10 CFU/mL)	Valnemulin (log10 CFU/mL)
10^5^	24	2.97	1.79	2.45	2.33	2.14
	36	3.55	3.04	2.77	2.89	2.89
	48	3.66	3.56	3.58	3.45	3.49
10^7^	24	2.40	1.77	3.96	3.46	1.83
	36	3.12	2.93	4.06	3.76	2.04
	48	4.05	4.10	4.32	4.42	2.26
10^9^	24	3.33	2.65	0.79	1.73	1.12
	36	3.71	3.07	3.09	3.18	3.85
	48	5.27	5.16	4.22	4.93	4.66

With exposure of 10^5^, 10^7^ and 10^9^ CFU/mL of *M*. *gallisepticum* to the MIC_99_ drug concentrations, and the survival colonies were counted on drug-free plates. The log10 (CFU/mL) reduction of *M*. *gallisepticum* count from 24 to 48 h is expressed in positive value, and data are persented as means of triplicates.

**Table 5:** The reduction of *M*. *gallisepticum* growth for three different inoculum sizes based on the measured MPC concentrations.

Inoculum size (CFU/mL)	Time (h)	Danofloxacin (log10 CFU/mL)	Doxycycline (log10 CFU/mL)	Tilmicosin (log10 CFU/mL)	Tylvalosin (log10 CFU/mL)	Valnemulin (log10 CFU/mL)
10^5^	24	3.66	3.66	3.66	3.66	3.01
	36	3.66	3.66	3.66	3.66	3.28
	48	3.66	3.66	3.66	3.66	3.46
10^7^	24	5.58	3.24	5.58	5.58	3.08
	36	5.58	4.44	5.58	5.58	3.66
	48	5.58	5.23	5.58	5.58	4.88
10^9^	24	6.79	3.44	4.17	5.45	1.74
	36	7.05	4.21	5.25	5.81	4.31
	48	7.05	5.64	5.70	6.03	4.91

With exposure of 10^5^, 10^7^ and 10^9^ CFU/mL of *M*. *gallisepticum* to the MPC drug concentrations, and the survival colonies were counted on drug-free plates. The log10 (CFU/mL) reduction of *M*. *gallisepticum* counts from 24 to 48 h is expressed in positive value, and data are persented as means of triplicates.

With exposure of 10^5^ CFU/mL of *M*. *gallisepticum* to the MIC_99_ drug concentrations, tylvalosin (3.45) had the slowest rate of reduction in viable organisms and danofloxacin (3.66) had the highest rate of reduction at 48h. With exposure of 10^5^ CFU/mL inoculum to the MPC drug concentrations, valnemulin (3.46) had the slowest rate of reduction in viable organisms at 48 h and danofloxacin (3.66) had the highest rate of reduction at 6 h.

With exposure of 10^7^ CFU/mL of *M*. *gallisepticum* to the MIC_99_ drug concentrations, valnemulin (2.26) had the slowest rate of reduction in viable organisms and tylvalosin (4.42) had the highest rate of reduction at 48h. With exposure of 10^7^ CFU/mL inoculum to the MPC drug concentrations, valnemulin (4.88) had the slowest rate of reduction in viable organisms at 48 h and danofloxacin (5.58) had the highest rate of reduction at 9 h.

**Fig 2:**
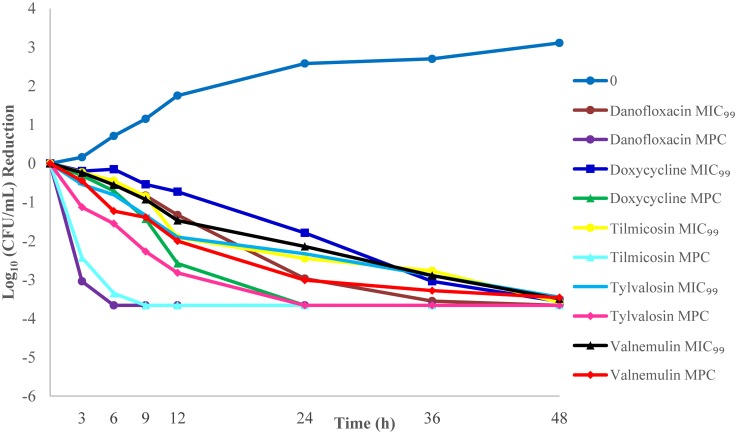
*M*. *gallisepticum* killing curves at the minimal concentration that inhibits colony formation by 99% (MIC_99_) and mutant prevention concentration (MPC) for an inoculum of 10^5^ CFU/mL.

**Fig 3:**
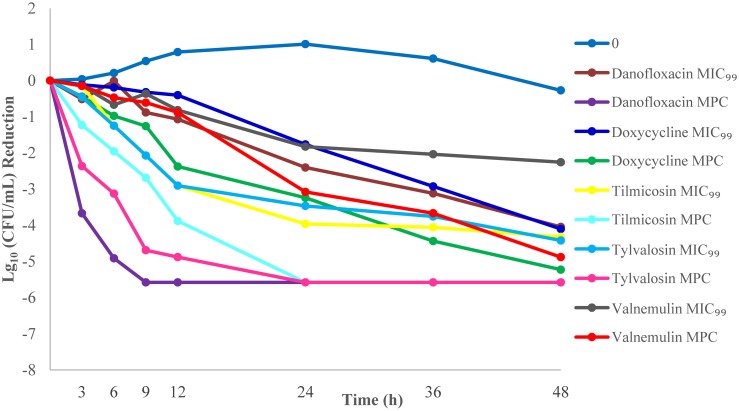
*M*. *gallisepticum* killing curves at the minimal concentration that inhibits colony formation by 99% (MIC_99_) and mutant prevention concentration (MPC) for an inoculum of 10^7^ CFU/mL.

**Fig 4:**
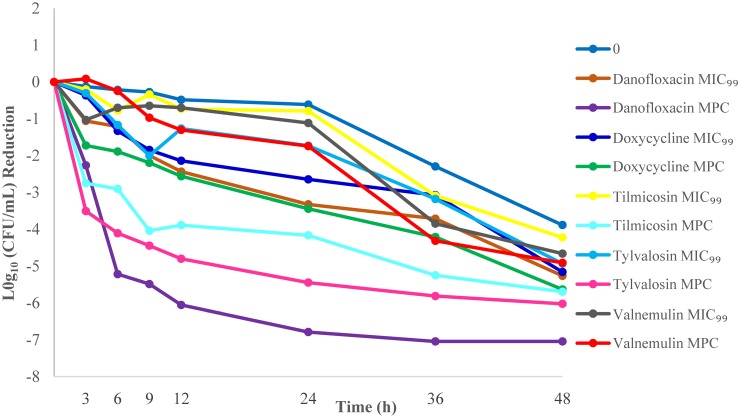
*M*. *gallisepticum* killing curves at the minimal concentration that inhibits colony formation by 99% (MIC_99_) and mutant prevention concentration (MPC) for an inoculum of 10^9^ CFU/mL.

With exposure of 10^9^ CFU/mL of *M*. *gallisepticum* to the MIC_99_ drug concentrations, tilmicosin (4.22) had the slowest rate of reduction in viable organisms and danofloxacin (5.27) had the highest rate of reduction at 48h. With exposure of 10^9^ CFU/mL inoculum to the MPC drug concentrations, valnemulin (4.91) had the slowest rate of reduction in viable organisms at 48 h and danofloxacin (7.05) had the highest rate of reduction at 36 h. Overall, killing was lowest and less complete with valnemulin at MPC concentrations over all inoculum sizes. Danofloxacin had the highest log10 (CFU/mL) reduction at MPC concentrations over all inoculum sizes (Figs 2–4).

## Discussion

This manuscript represents the first report of MIC_99_ and MPC determination of *M*. *gallisepticum* against the commonly used antimicrobial agents (danofloxacin, doxycycline, tilmicosin, tylvalosin and valnemulin). While some drugs such as tetracyclines and macrolides in common are used worldwide to control mycoplasma infections in poultry and other production animals, other antimicrobials such as fluoroquinolones are not necessarily recommended. Many fluoroquinolones are critically important antimicrobials, and should be reserved to treat human infections. A prudent use of veterinary antibiotics, particularly in intensive farming industries, is recommended. The results of our study demonstrated that the MPC could be determined in *M*. *gallisepticum*. In order to reduce the emergence of antimicrobial resistance in *M*. *gallisepticum*, the mutant selection window can be used as a framework for the design of antimicrobial therapies in *M*. *gallisepticum* infections.

Mycoplasmas are dependent on outside sources of precursor molecules for macromolecular syntheses. The serum component of mycoplasma media is essentially the only source for cholesterol and long-chain fatty acids[20, 21]. Horse serum has been used most frequently in mycoplasma media. However, our results indicated that it is preferable to culture them in the media containing swine serum on the basis of the improved morphology and the reproducibility of *M*. *gallisepticum* colonies on agar plates. The horse serum source may also affect colony growth. It was difficult for us to obtain a steady source of commercial horse serum from Guangdong province. Moreover, lipoprotein concentrations in animal sera may also vary by diet. A published article reported that very low density lipoproteins (VLDL) in swine plasma (43.5 mg/100mL) was higher than other plasma (33.3 mg/100mL in horse plasma and 10 mg/100mL in bovine plasma)[22]. Hence, VLDL may be the main active ingredient for culturing *M*. *gallisepticum*.

MIC values in the present study using the liquid method were similar to previous studies [13, 18, 23, 24]. For the five antimicrobial agents, the MIC determination for tilmicosin was very sensitive to inoculum effects. The MIC values of tilmicosin obtained from the high inoculum size (10^7^ CFU/mL) were four and two times higher than those obtained from the low inoculum size (10^5^ CFU/mL) using the liquid MIC method and the solid agar method respectively. In an earlier report [25], increased MIC with increasing inoculum size were also observed when *S*. *aureus* was applied to agar containing various concentrations of daptomycin. Except for doxycycline, the MIC values of the other antimicrobials obtained from the solid agar method were two to four times higher than those determined using the liquid MIC method for each inoculum size. The 8-fold greater MICs of doxycycline with the agar method may be the result of a more rapid decomposition rate of this drug in agar during the extended incubation period[26, 27].

MPC is defined as the MIC of the least susceptible single-step mutant. MPC measurements may not only apply to fluoroquinolone compounds because spontaneous chromosomal point mutations have been observed with fluoroquinolones, but also β–lactams, macrolides and aminoglycosides rely on the acquisition of transmissible foreign DNA containing respective resistance genes. The difference is the population analysis curve is higher for plasmid borne resistance because it enters a population of bacteria at an elevated frequency. In the case of *M*. *gallisepticum*, fluoroquinolone-resistant mutants have been found in the quinolone resistance-determining regions of the four target genes encoding DNA gyrase and topoisomerase IV[28]. There is no published study on tetracycline resistance in *M*. *gallisepticum* although resistance acquired *via tetM* transfer has been seen in other mycoplasmas[29]. *M*. *gallisepticum* could acquire resistance to tilmicosin and valnemulin through 23S rDNA point mutations[30, 31]. Despite the mechanism of reduced susceptibility and antimicrobial drug class, MPC defines antimicrobial drug concentrations that block the growth of resistant sub-populations from high density bacterial populations. It does not mean to resolve all the current resistance issues.

Although the MSW dimensions are unique for each drug-pathogen combination, it is a useful value in reporting what clinical regiments pose the least risk in enrichment for resistant mutants [32]. For *M*. *gallisepticum* strain S6, the reported danofloxacin and doxycycline concentrations in chicken fell partially within the MSW after oral administration at doses of 5 and 10 mg/kg. The C_max_ of danofloxacin and doxycycline were from 0.47 to 0.53, and 3.07 to 4.47 μg/mL. The corresponding MIC_99_ and MPC levels were 0.10 and 2.40; 1.10 and 2.46 μg/mL, respectively[33–36]. While the reported pharmacokinetic measurements of tilmicosin, tylvalosin and valnemulin after oral administration at the dose of 30, 20 and 10 mg/kg showed that concentrations of these agents were far more than the MPC determined *in vitro* [37–39], revealing that the pharmacokinetic profile were above the MSW thoroughly. Consequently, it might be difficult for tilmicosin tylvalosin and valnemulin to select the resistance mutant. In some previous studies, drug resistance increased when the pharmacokinetic profile fell inside the selection window [5, 40–43]. For example, in simulated drug dosing in the MSW, mutant subpopulations were enriched in the presence of the drug with decreased susceptibility when testing fluoroquinolones against either *S*. *pneumonia* or *S*. *aureus* [40]. Levofloxacin susceptibility of *S*. *aureus* decreased and mutant subpopulations emerged when drug concentrations fell inside the selection window. Considering the pharmacokinetics, we could speculate that tilmicosin, tylvalosin and valnemulin are less likely to select resistant mutants than danofloxacin and doxycycline.

The bactericidal effects of the antimicrobials were also tested. In this work, killing was poorer at MIC_99_ drug concentrations than MPC drug concentrations, particularly against higher inoculum sizes. Killing at the MPC demonstrated that valnemulin had the slowest rate of reduction in viable organisms, whereas danofloxacin had the highest. The killing kinetics seen here for valnemulin against *M*. *gallisepticum* are consistent with an earlier report[24]. In their study, time-killing studies were conducted using a mycoplasma inoculum of 10^6^ CCU/mL exposed to the concentrations from 0.25-fold to 32-fold of the MIC and the Log10 (CFU/mL) reduction in viable cells was recorded from 0 to 24 h after drug exposure. The authors reported a mycoplasmacidal effect after 24 h exposure of valnemulin (≥ 3 log10 (CFU/mL) reduction) at 7× MIC (0.0053 mg/L). Results from the current report using mycoplasma inocula of 10^5^−10^7^ CFU/mL are similar to the values reported by Xiao et al[24]. Based on exposure at the MPC (0.0051 mg/L), a mycoplasmacidal effect was observed following 24 h of drug exposure. The limitations of this study were that our *in vitro* measurements were conducted in an ideal environment where drug concentrations could be held constant over the duration of measurement. We are in the process of validating these results *in vivo*.

## Conclusion

This study was the first to determine the MSW of danofloxacin, doxycycline, tilmicosin, tylvalosin and valnemulin against *M*. *gallisepticum in vitro*. The abilities of these drugs in terms of rate and completeness of killing were clearly different. Such observations are valuable in choosing antimicrobials for therapy. Considering the pharmacokinetics, we could speculate that tilmicosin, tylvalosin and valnemulin are less likely to select resistant mutants than danofloxacin and doxycycline. In addition, the current study also represents the first report of killing of *M*. *gallisepticum* of these five antimicrobial drugs with high-density bacterial populations.

## Supporting Information

S1 Table Minimum inhibitory concentrations for danofloxacin, doxycycline, tilmicosin, tylvalosin and valnemulin against *M*. *gallisepticum* strain S6 in artificial medium using the liquid method with inoculum sizes of 10^5^,10^6^ and 10^7^ CFU/mL. The experiment were performed in triplicate and conducted on three days.
(DOCX)

S2 Table Minimum inhibitory concentrations for danofloxacin, doxycycline, tilmicosin, tylvalosin and valnemulin against *M*. *gallisepticum* strain S6 in artificial medium using solid agar method with inoculum sizes of 10^5^,10^6^ and 10^7^ CFU/mL. The experiments were performed in triplicate and conducted on three days.
(DOCX)

S3 Table Effects of different serum types on *M*. *gallisepticum* growth on agar plates. The experiment were performed in triplicate and conducted on three days.
(DOCX)

S4 Table The results of three different pretreatment methods for *M*. *gallisepticum* enrichment. The experiment were performed in triplicate and conducted on three days.
(DOCX)

S5 Table The reduction of *M*. *gallisepticum* growth for three different inoculum sizes based on the measured MIC_99_ concentrations. With exposure of 10^5^, 10^7^ and 10^9^ CFU/mL of *M*. *gallisepticum* to the MIC_99_ drug concentrations, and the survival colonies were counted on drug-free plates. The log10 (CFU/mL) reduction of *M*. *gallisepticum* count from 24 to 48 h is expressed in positive value, and data are presented as means of triplicates.
(DOCX)

S6 Table The reduction of M. gallisepticum growth for three different inoculum sizes based on the measured MPC concentrations. With exposure of 10^5^, 10^7^ and 10^9^ CFU/mL of *M*. *gallisepticum* to the MPC drug concentrations, and the survival colonies were counted on drug-free plates. The log10 (CFU/mL) reduction of *M*. *gallisepticum* counts from 24 to 48 h is expressed in positive value, and data are persented as means of triplicates.
(DOCX)
