# Supplementary material for: Determination of the Mutant Selection Window and Evaluation of the Killing of Mycoplasma gallisepticum by Danofloxacin, Doxycycline, Tilmicosin, Tylvalosin and Valnemulin
Source: PLoS One. 2017 Jan 4;12(1):e0169134. doi: 10.1371/journal.pone.0169134 (PMC5215565; doi:10.1371/journal.pone.0169134)
Supplement: S1 Table — The experiment were performed in triplicate and conducted on three days. (DOCX) [file pone.0169134.s001.docx]

**Supporting Information**

Table 1. Minimum inhibitory concentrations for danofloxacin, doxycycline, tilmicosin,

tylvalosin and valnemulin against *M. gallisepticum* strain S6 in artificial medium using the liquid method with inoculum sizes of 10^5^,10^6^ and 10^7^ CFU/mL. The experiment were performed in triplicate and conducted on three days.

| Inoculum size  (CFU/mL) | Danofloxacin  (mg/L) | Doxycycline  (mg/L) | Tilmicosin  (mg/L) | Tylvalosin  (mg/L) | | Valnemulin  (mg/L) |
| --- | --- | --- | --- | --- | --- | --- |
| 10^5^ | 0.038 | 0.15 | 0.019 | 0.013 | 0.0013 | |
| 10^6^  10^7^ | 0.05 | 0.15 | 0.038 | 0.019 | 0.0013 | |
|  | 0.075 | 0.15 | 0.075 | 0.025 | 0.0013 | |
