# Supplementary material for: Determination of the Mutant Selection Window and Evaluation of the Killing of Mycoplasma gallisepticum by Danofloxacin, Doxycycline, Tilmicosin, Tylvalosin and Valnemulin
Source: PLoS One. 2017 Jan 4;12(1):e0169134. doi: 10.1371/journal.pone.0169134 (PMC5215565; doi:10.1371/journal.pone.0169134)
Supplement: S3 Table — The experiment were performed in triplicate and conducted on three days. (DOCX) [file pone.0169134.s003.docx]

**Supporting Information**

Table 3. Effects of different serum types on *M. gallisepticum* growth on agar plates. The experiment were performed in triplicate and conducted on three days.

| Serum | First time  Log_10_ CFU | | | Second time  Log_10_ CFU | | | Third time  Log_10_ CFU | | |
| --- | --- | --- | --- | --- | --- | --- | --- | --- | --- |
| Swine serun | 8.78 | 8.76 | 8.68 | 8.79 | 8.71 | 8.74 | 8.80 | 8.76 | 8.7 |
| Horse serum | 8.82 | 8.53 | 8.66 | 8.72 | 8.61 | 8.83 | 8.70 | 8.72 | 8.40 |
| Bovine serum | 8.49 | 8.40 | 8.57 | 8.51 | 8.48 | 8.43 | 8.54 | 8.46 | 8.49 |
| Mixed serum | 8.83 | 8.72 | 8.71 | 8.46 | 8.65 | 8.70 | 8.71 | 8.52 | 8.45 |
