# Supplementary material for: Determination of the Mutant Selection Window and Evaluation of the Killing of Mycoplasma gallisepticum by Danofloxacin, Doxycycline, Tilmicosin, Tylvalosin and Valnemulin
Source: PLoS One. 2017 Jan 4;12(1):e0169134. doi: 10.1371/journal.pone.0169134 (PMC5215565; doi:10.1371/journal.pone.0169134)
Supplement: S4 Table — The experiment were performed in triplicate and conducted on three days. (DOCX) [file pone.0169134.s004.docx]

**Supporting Information**

Table 4. The results of three different pretreatment methods for *M. gallisepticum* enrichment. The experiment were performed in triplicate and conducted on three days.

| Method | First time  Log_10_ CFU | | | Second time  Log_10_ CFU | | | Third time  Log_10_ CFU | | |
| --- | --- | --- | --- | --- | --- | --- | --- | --- | --- |
| 1 | 9.49 | 9.54 | 9.67 | 9.08 | 9.32 | 9.60 | 9.36 | 9.54 | 9.23 |
| 2 | 9.38 | 9.82 | 9.89 | 9.83 | 9.23 | 9.46 | 9.15 | 9.59 | 9.72 |
| 3 | 10.40 | 10.43 | 10.62 | 10.63 | 10.45 | 10.57 | 10.59 | 10.43 | 10.71 |
