# Supplementary material for: Determination of the Mutant Selection Window and Evaluation of the Killing of Mycoplasma gallisepticum by Danofloxacin, Doxycycline, Tilmicosin, Tylvalosin and Valnemulin
Source: PLoS One. 2017 Jan 4;12(1):e0169134. doi: 10.1371/journal.pone.0169134 (PMC5215565; doi:10.1371/journal.pone.0169134)
Supplement: S5 Table — With exposure of 105, 107 and 109 CFU/mL of M. gallisepticum to the MIC99 drug concentrations, and the survival colonies were counted on drug-free plates. The log10 (CFU/mL) reduction of M. gallisepticum count from 24 to 48 h is expressed in positive value, and data are presented as means of triplicates. (DOCX) [file pone.0169134.s005.docx]

**Supporting Information**

Table 5. The reduction of *M. gallisepticum* growth for three different inoculum sizes based on the measured MIC_99_ concentrations.

| Inoculum size  (CFU/mL) | Time  (h) | Danofloxacin  (log10 CFU/mL) | Doxycycline  (log10 CFU/mL) | Tilmicosin  (log10 CFU/mL) | Tylvalosin  (log10 CFU/mL) | | Valnemulin   (log10 CFU/mL) |
| --- | --- | --- | --- | --- | --- | --- | --- |
| 10^5^ | 3 | 0.23 | 0.20 | 0.23 | 0.54 | 0.24 | |
|  | 6 | 0.55 | 0.15 | 0.45 | 0.81 | 0.55 | |
|  | 9 | 0.83 | 0.54 | 0.85 | 1.34 | 0.93 | |
|  | 12 | 1.33 | 0.73 | 1.91 | 1.9 | 1.47 | |
|  | 24 | 2.97 | 1.79 | 2.45 | 2.33 | 2.14 | |
|  | 36 | 3.55 | 3.04 | 2.77 | 2.89 | 2.89 | |
|  | 48 | 3.66 | 3.56 | 3.58 | 3.45 | 3.49 | |
|  | 3 | 0.51 | 0.12 | 0.15 | 0.46 | 0.14 | |
|  | 6 | 0.01 | 0.19 | 1.25 | 1.25 | 0.67 | |
|  | 9 | 0.88 | 0.32 | 2.07 | 2.07 | 0.37 | |
| 10^7^ | 12 | 1.07 | 0.40 | 2.9 | 2.9 | 0.82 | |
|  | 24 | 2.40 | 1.77 | 3.96 | 3.46 | 1.83 | |
|  | 36 | 3.12 | 2.93 | 4.06 | 3.76 | 2.04 | |
|  | 48 | 4.05 | 4.10 | 4.32 | 4.42 | 2.26 | |
|  | 3 | 1.06 | 0.37 | 0.20 | 0.30 | 1.03 | |
|  | 6 | 1.21 | 1.33 | 0.78 | 1.17 | 0.70 | |
|  | 9 | 2.00 | 1.85 | 0.34 | 2.00 | 0.65 | |
| 10^9^ | 12 | 2.44 | 2.14 | 0.73 | 1.27 | 0.70 | |
|  | 24 | 3.33 | 2.65 | 0.79 | 1.73 | 1.12 | |
|  | 36 | 3.71 | 3.07 | 3.09 | 3.18 | 3.85 | |
|  | 48 | 5.27 | 5.16 | 4.22 | 4.93 | 4.66 | |

With exposure of 10^5^, 10^7^ and 10^9^ CFU/mL of *M. gallisepticum* to the MIC_99_ drug concentrations, and the survival colonies were counted on drug-free plates. The log10 (CFU/mL) reduction of *M. gallisepticum* count from 24 to 48 h is expressed in positive value, and data are persented as means of triplicates.

Table 6. The reduction of *M. gallisepticum* growth for three different inoculum sizes based on the measured MPC concentrations.

| Inoculum size  (CFU/mL) | Time  (h) | Danofloxacin  (log10 CFU/mL) | Doxycycline  (log10 CFU/mL) | Tilmicosin  (log10 CFU/mL) | Tylvalosin  (log10 CFU/mL) | Valnemulin   (log10 CFU/mL) |
| --- | --- | --- | --- | --- | --- | --- |
|  | 3 | 3.04 | 0.31 | 2.43 | 1.13 | 0.45 |
|  | 6 | 3.66 | 0.71 | 3.35 | 1.55 | 1.23 |
|  | 9 | 3.66 | 1.43 | 3.66 | 2.27 | 1.39 |
| 10^5^ | 12 | 3.66 | 2.58 | 3.66 | 2.82 | 2 |
|  | 24 | 3.66 | 3.66 | 3.66 | 3.66 | 3.01 |
|  | 36 | 3.66 | 3.66 | 3.66 | 3.66 | 3.28 |
|  | 48 | 3.66 | 3.66 | 3.66 | 3.66 | 3.46 |
|  | 3 | 3.67 | 0.44 | 1.23 | 2.36 | 0.15 |
|  | 6 | 4.91 | 0.98 | 1.96 | 3.13 | 0.47 |
|  | 9 | 5.58 | 1.26 | 2.69 | 4.69 | 0.61 |
| 10^7^ | 12 | 5.58 | 2.37 | 3.88 | 4.88 | 0.88 |
|  | 24 | 5.58 | 3.24 | 5.58 | 5.58 | 3.08 |
|  | 36 | 5.58 | 4.44 | 5.58 | 5.58 | 3.66 |
|  | 48 | 5.58 | 5.23 | 5.58 | 5.58 | 4.88 |
|  | 3 | 2.27 | 1.73 | 2.75 | 3.51 | 0.08 |
|  | 6 | 5.22 | 1.89 | 2.91 | 4.11 | 0.25 |
|  | 9 | 5.49 | 2.2 | 4.04 | 4.45 | 0.97 |
| 10^9^ | 12 | 6.06 | 2.56 | 3.89 | 4.8 | 1.30 |
|  | 24 | 6.79 | 3.44 | 4.17 | 5.45 | 1.74 |
|  | 36 | 7.05 | 4.21 | 5.25 | 5.81 | 4.31 |
|  | 48 | 7.05 | 5．64 | 5.70 | 6.03 | 4.91 |

Exposure of 10^5^, 10^7^ and 10^9^ CFU/mL of *M. gallisepticum* to the MPC drug concentrations, and the survival colonies were counted on drug-free plates. The log10 (CFU/mL) reduction of *M. gallisepticum* counts from 24 to 48 h is expressed in positive value, and data are persented as means of triplicates.
